# Culture of haploid blastocysts in FGF4 favors the derivation of epiblast stem cells with a primed epigenetic and transcriptional landscape

**DOI:** 10.1038/s41598-018-29074-6

**Published:** 2018-07-17

**Authors:** Runsheng He, Benjamin L. Kidder

**Affiliations:** 1 Department of Oncology, Wayne State University School of Medicine, Detroit, MI USA; 2 Karmanos Cancer Institute, Wayne State University School of Medicine, Detroit, MI USA

## Abstract

Pluripotent stem cells within the inner cell mass and epiblast of mammalian embryos have the capacity to form all lineages in the adult organism, while multipotent trophoblast stem (TS) cells derived from the trophectoderm are capable of differentiating into fetal lineages of the placenta. While mouse embryonic stem (ES) cells and epiblast stem cells (EpiSCs) exhibit distinct expression patterns and utilize distinct external signaling pathways for self-renewal, because mouse EpiSCs resemble human ES cells they are a useful model to investigate mechanisms of human ES cell self-renewal and differentiation. Recent studies have shown that haploid embryos and ES cells can be generated from chemically-activated unfertilized mouse oocytes. However, it is unclear whether EpiSCs or TS cells can be derived from haploid embryos. Here, we describe the derivation of EpiSCs from haploid blastocyst-stage embryos using culture conditions that promote TS cell self-renewal. Maternal (parthenogenetic/gynogenetic) EpiSCs (maEpiSCs) functionally and morphologically resemble conventional EpiSCs. Established maEpiSCs and conventional EpiSCs are diploid and exhibit a normal number of chromosomes. Moreover, global expression analyses and epigenomic profiling revealed that maEpiSCs and conventional EpiSCs exhibit similarly primed transcriptional programs and epigenetic profiles, respectively. Altogether, our results describe a useful experimental model to generate EpiSCs from haploid embryos, provide insight into self-renewal mechanisms of EpiSCs, and suggest that FGF4 is not sufficient to derive TS cells from haploid blastocyst-stage embryos.

## Introduction

Pluripotent stem cells originating from the inner cell mass (ICM) of pre-implantation blastocysts and epiblast stem cells (EpiSCs) derived from the epiblast of postimplantation embryos or pre-implantation embryos have the capacity to differentiate into cell types of the three embryonic germ layers. Mouse ES cells and EpiSCs are distinct pluripotent states which have conventionally been isolated from pre- and post-implantation embryos, respectively^1–4^. Moreover, recent work has demonstrated that EpiSCs can be derived from preimplantation embryos^5^. Mouse EpiSCs and human ES cells can both be maintained in culture indefinitely by activin/nodal and FGF signaling pathways^1,4,6^. Because human ES cells resemble mouse EpiSCs, understanding the molecular framework of mouse EpiSCs, including the transcriptional and epigenetic landscapes that promote self-renewal, may aid in our understanding of mechanisms of human ES cell self-renewal. Mouse trophoblast stem (TS) cells, which also originate from preimplantation stage embryos, can be cultured indefinitely in the presence of activin/nodal and FGF signaling pathways^7^. TS cells derived from the outer trophectoderm (TE) layer of the blastocyst are capable of differentiating into trophoblast (extraembryonic ectoderm) cell types following implantation^8,9^. The common signaling pathways that support self-renewal of pluripotent human ES cells, mouse EpiSCs and multipotent TS cells suggest that divergent fates of preimplantation-stage cells can be sustained in culture under similar conditions.

Here, we investigated whether culture of mouse haploid blastocyst-stage embryos generated from chemically-activated oocytes favors a pluripotent EpiSC or multipotent TS cell fate. While we did not observe the formation of TS cells from haploid blastocyst-stage embryos, we show that maternal (parthenogenetic/gynogenetic) EpiSCs (maEpiSCs) can be generated following culture of haploid mouse preimplantation-stage embryos in FGF4-culture conditions. Transcriptome analysis demonstrated that maEpiSCs display a similar gene expression landscape relative to conventional EpiSCs. Genome-wide epigenomic profiling also showed similar histone modification profiles between maEpiSCs and conventional EpiSCs. Moreover, maEpiSCs are capable of differentiating *in vitro* and *in vivo*. Overall, our results demonstrate the ability to generate maEpiSCs from haploid preimplantation-stage embryos.

## Results

### Generation of EpiSCs from haploid mouse blastocysts

**Figure 1:**
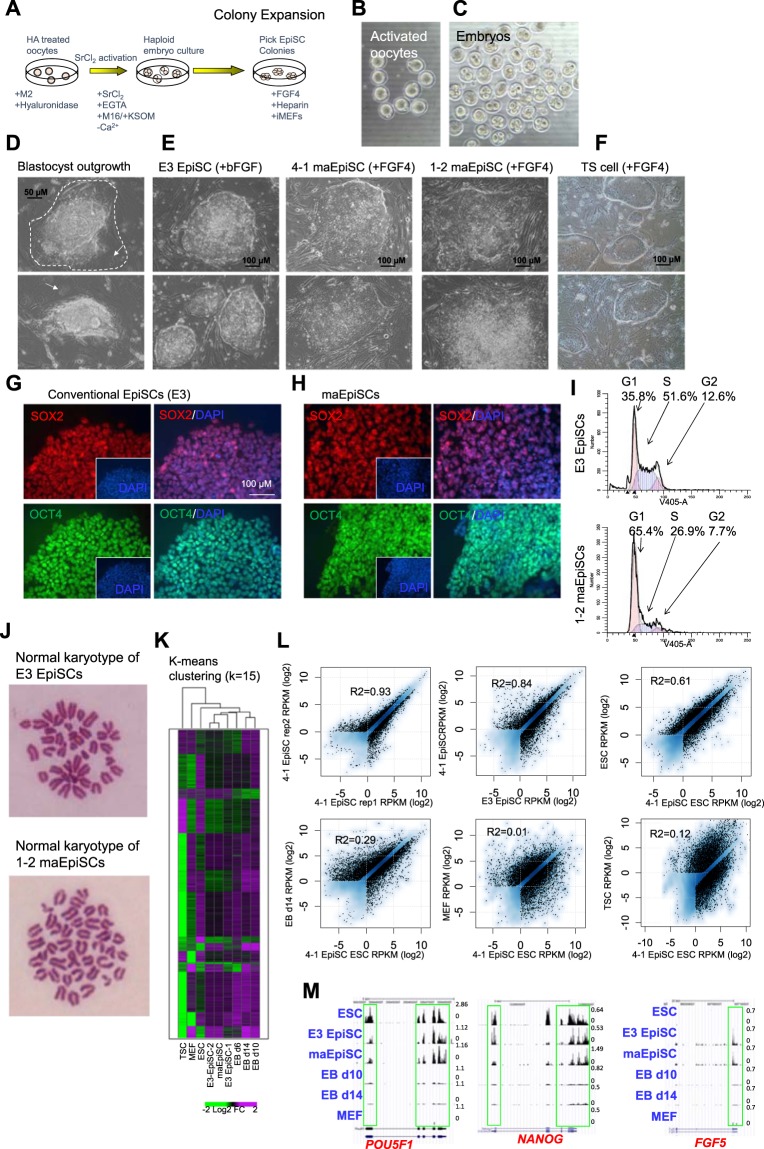
Derivation of Epiblast stem cells (EpiSCs) from haploid mouse embryos. (**A**) Experimental design schematic. Superovulated oocytes were activated in SrCl_2_-containing media and (**B**) activated oocytes were cultured to the (**C**) two-cell stage and subsequently to blastocyst-stage embryos. Blastocysts were cultured on iMEFs in medium containing FGF4 and EpiSC-like colonies were picked and expanded. (**D**) Bright-field microscopy of blastocyst outgrowths after 96 hrs. Arrow highlights trophectoderm (TE) cells, and dotted line outlines boundary of TE outgrowth. (**E**) Bright-field microscopy of conventional EpiSCs (E3; left panels) cultured in the presence of bFGF and two representative maternal (parthenogenetic/gynogenetic) EpiSCs (maEpiSCs; right panels) cultured in the presence of FGF4. (**F**) Bright-field microscopy of TS cells, derived from normal diploid blastocysts, and cultured in the presence of FGF4. (**G,H**) Immunofluorescence analysis of SOX2 and OCT4 in (**G**) conventional EpiSCs and (**H**) maEpiSCs. Nuclei were stained with Dapi. (**I**) Dapi cell cycle staining of conventional EpiSCs and maEpiSCs. (**J**) Metaphase spread Giesma staining and chromosome counts show that conventional EpiSCs and maEpiSCs have a normal karyotype. (**K**) Clustering analysis (K-means) of RNA-Seq data. Clustering was performed according to k-means on differentially expressed (>two-fold) genes. (**L**) RNA-Seq expression analysis, represented on a scatter plot, of two maEpiSC biological replicates, maEpiSCs and conventional EpiSCs (E3, top middle), ES cells (top right), MEFs (bottom right), and differentiated ES cells (embryoid body (EB) day 14; bottom left). Differentially expressed genes (Log2 adjusted) are shown (RPKM > 1, FC > two-fold). (**M**) UCSC browser views of RNA-Seq data.

Because FGF4 has previously been shown to support the self-renewal of both mouse TS cells and EpiSCs^7,10^, we investigated whether FGF4-culture conditions favors the generation of EpiSCs or TS cells from haploid mouse preimplantation-stage embryos. To this end, we generated haploid embryos by chemically activating unfertilized mouse oocytes isolated from superovulated C57Bl/6 J female mice using strontium chloride as previously described^11^ (Fig. 1A). Parthenogenetically activated oocytes (Fig. 1B) were cultured in M16 media, where they developed into 2-cell embryos the following day (Fig. 1C). After culture in M16 media to the blastocyst stage, embryos were cultured in media containing FGF4 (see methods) on mitotically inactivated mouse embryonic fibroblasts (iMEFs), and outgrowths were subsequently observed (Fig. 1D). While we observed trophoblast giant-like cells from the outgrowths of haploid blastocysts (Fig. 1D), we did not observe the formation of TS cell colonies, suggesting that derivation of TS cells may require a diploid genome. The potential inability of a haploid genome to support TS cell self-renewal is in alignment with the rapid diploidization of haploid ES cells observed following induction of differentiation^12^. It is possible that the haploid genome state is unstable in lineage-committed cells, such as in TS cells, which are derived from the trophectoderm, which is the first lineage to emerge during development. However, we were able to generate several EpiSC lines using this strategy (Fig. 1E). Individual maternal (parthenogenetic/gynogenetic) EpiSCs (maEpiSCs) were picked and expanded for further evaluation (Fig. 1E). The morphology of conventional EpiSCs (E3)^13^ (Fig. 1E, left) cultured in media containing bFGF (see methods) was similar to representative maEpiSC cell lines (Fig. 1E, right) cultured in media containing FGF4. While we did not observe the formation of TS cell colonies from haploid blastocysts, culture of normal diploid blastocysts, generated from fertilized oocytes, in media containing FGF4 was suitable for establishment of TS cells (Fig. 1F). Immunofluorescence staining confirmed that conventional EpiSCs (Fig. 1G) and maEpiSCs cells (Fig. 1H) express the pluripotency markers SOX2 and OCT4. In addition, maEpiSCs exhibited an altered cell cycle pattern relative to conventional EpiSCs (Fig. 1I). For example, 35.8% of conventional EpiSCs and 65.4% of maEpiSCs were in the G1 phase of the cell cycle, 51.6% of conventional EpiSCs and 26.9% of maEpiSCs were in the S phase, and 12.6% and 7.7% were in the G2 phase (Fig. 1I). These findings likely reflect a decreased rate of proliferation for maEpiSCs relative to conventional EpiSCs. Moreover, chromosome counting analyses demonstrated that established maEpiSCs and conventional EpiSCs are diploid and exhibit a normal karyotype (40 chromosomes) (Fig. 1J). Combined, these results demonstrate that maEpiSCs can be derived from haploid blastocysts.

### maEpiSCs have a similar gene expression profile compared to conventional EpiSCs

To evaluate whether maEpiSCs exhibit an EpiSC-like transcriptional profile we utilized RNA-Seq. The transcriptome of a representative maEpiSC cell line was interrogated relative to conventional EpiSCs, ES cells, and embryoid body (EB) differentiated (day 10 and day 14) ES cells, mouse embryonic fibroblasts (MEFs), and TS cells. Like EpiSCs, ES cells also express elevated levels of OCT4, and are pluripotent. ES cells represent another control for a pluripotent cell type, while EBs and MEFs represent differentiated cells. Hierarchical clustering (HCA) and k-means clustering were used to identify variations in patterns of gene expression. These results showed that maEpiSCs and conventional EpiSCs clustered closer together relative to ES cells, TS cells, and differentiated EBs and MEF cells (Fig. 1K). Moreover, the expression profile of maEpiSCs was more similar to conventional EpiSCs (E3) relative to ES cells, TS cells, or differentiated cells (EBs and MEFs) (Fig. 1L). Custom RNA-Seq UCSC genome browser views showed that maEpiSCs and conventional EpiSCs express the pluripotency-factors *Nanog*, *Pou5f1*, and the EpiSC marker *Fgf5* (Fig. 1M), while MEFs and differentiated EBs (day 10 and day 14) did not express these genes.

### Comparable epigenetic landscape of maEpiSCs and conventional EpiSCs

**Figure 2:**
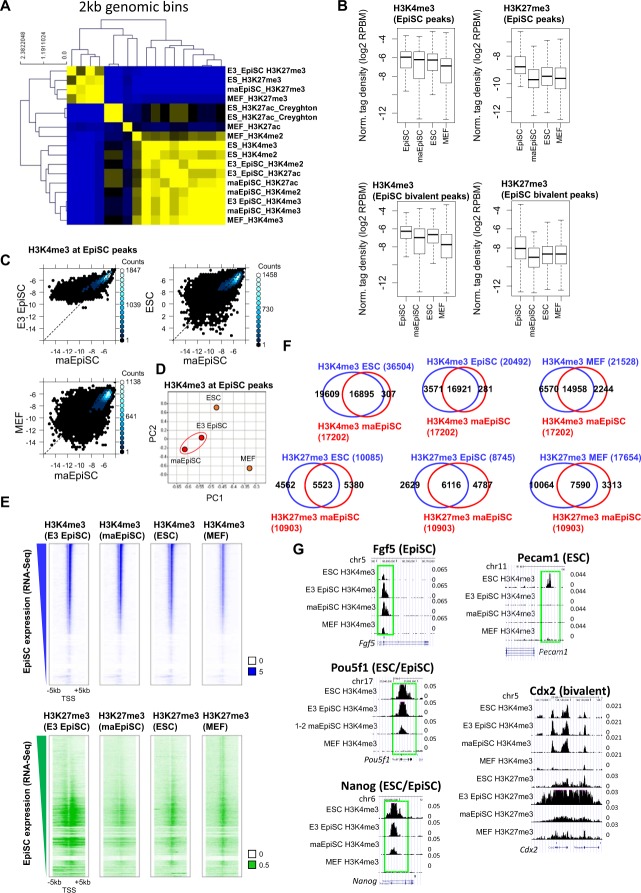
Epigenomic profiling of maEpiSCs. (**A**) Heat map of correlation matrix of histone modification, epigenetic regulator, and transcriptional regulator densities at 2 kb genomic intervals between maEpiSCs, conventional EpiSCs, MEFs, and ESCs. Pair-wise affinities between data generated in this study and public data are shown in a heat map^17,39–41^. Pair-wise affinity values were generated using AutoSOME^42^. (**B**) H3K4me3 and H3K27me3 densities in conventional EpiSCs (E3), maEpiSC, ES cells, and MEFs are shown in boxplots at conventional EpiSC H3K4me3-peaks (top) and at H3K4me3/H3K27me3 bivalent peaks (bottom). (**C**) H3K4me3 densities at EpiSC-peaks shown in scatter plots (reads per base per million reads (RPBM); log2 normalized tag density). (**D**) Evaluation of H3K4me3 densities in maEpiSCs, conventional EpiSCs, ESCs and MEFs using principal component analysis (PCA). (**E**) H3K4me3 and H3K27me3 densities represented as heat maps at transcriptional start site (TSS) regions. (**F**) Overlap between ChIP-enriched peaks in maEpiSCs and ESCs, conventional EpiSCs and MEFs are shown in Venn diagrams. (**G**) UCSC genome browser views of H3K27me3 and H3K4me3 ChIP-Seq data in maEpiSCs, conventional EpiSCs, ESCs, and MEFs.

Next, we investigated global profiles of histone modifications in maEpiSCs using ChIP-Seq and previously described methods^14–17^. By comparing maEpiSC histone modification profiles with histone modification patterns in conventional EpiSCs, ES cells, and MEFs, using 2 kb genomic bins, we found that genome-wide histone modification profiles of maEpiSCs are similar to conventional EpiSCs (E3) (Fig. 2A). Furthermore, boxplots reveal that H3K4me3 levels at conventional EpiSC peaks are overall similar between conventional EpiSCs and maEpiSCs (Fig. 2B, top left), while MEFs (GSE21271) and ESCs (GSE53087) displayed decreased levels at conventional EpiSC ChIP-Seq peaks (SICER-defined peaks, see methods). Moreover, while levels of H3K4me3 at H3K4me3/H3K27me3 bivalent chromatin regions in EpiSCs were similar between conventional EpiSCs, maEpiSCs, and ES cells (Fig. 2B, bottom left), levels of H3K27me3 at EpiSC bivalent peaks were lower in maEpiSCs relative to conventional EpiSCs. However, levels of H3K27me3 at EpiSC peaks were lower in maEpiSCs relative to conventional EpiSCs (Fig. 2B, top right).

While scatter plots demonstrated that maEpiSC H3K4me3 profiles were more similar to conventional EpiSCs relative to ES cells or MEFs (Fig. 2C), a comparison of conventional EpiSCs and maEpiSCs demonstrated that levels of H3K4me3 were lower in maEpiSCs at a subset of regions. PCA further confirmed that maEpiSCs are more similar to conventional EpiSCs (E3) relative to ES cells or MEFs (Fig. 2D). PCA revealed that maEpiSCs and conventional EpiSCs clustered closer to one another within the two-dimensional space, and along the PC1 and PC2 axes, relative to ES cells or MEFs. Heat maps also showed that conventional EpiSCs and maEpiSCs exhibited similar global profiles of H3K4me3 (Fig. 2E, top), where H3K4me3 levels were higher in maEpiSCs and conventional EpiSCs relative to ESCs and MEFs, at genes whose expression is elevated in EpiSCs. Moreover, while conventional EpiSCs, maEpiSCs, and ES cells exhibited relatively similar distributions of H3K27me3 (Fig. 2E, bottom), MEFs exhibited higher levels of H3K27me3 at genes whose expression is elevated in EpiSCs (Fig. 2E, bottom). In addition, our ChIP-Seq results show that 83% of regions containing H3K4me3-marks in conventional EpiSCs were also marked by H3K4me3 in maEpiSCs, whereas 46% of regions containing H3K4me3-marks in ES cells were also occupied by H3K4me3 in maEpiSCs (Fig. 2F), and 69% of regions containing H3K4me3-marks in MEFs were also occupied by H3K4me3 in maEpiSCs (Fig. 2F), suggesting that H3K4me3 levels in maEpiSCs are more similar to conventional EpiSCs than ES cells or MEFs. Also, 69% of regions containing H3K27me3 in conventional EpiSCs were also occupied by H3K27me3 in maEpiSCs (Fig. 2F, bottom). In contrast, 43% and 45% of regions containing H3K27me3 in MEFs and ES cells, respectively, were also occupied by H3K27me3 in maEpiSCs (Fig. 2F, bottom). These findings suggest that H3K27me3 profiles in maEpiSCs resemble conventional EpiSCs more than ES cells or MEFs.

Conventional EpiSCs and maEpiSCs both had elevated H3K4me3 levels at *Fgf5*, whereas ES cells and MEFs had low H3K4me3 levels at *Fgf5*, a gene whose expression is enriched in EpiSCs (Fig. 2G, top left), further showing that maEpiSCs epigenetically resemble conventional EpiSCs compared to MEFs or ES cells. Moreover, we observed H3K4me3 enrichment at pluripotency-related genes, including *Nanog* and *Pou5f1*, in UCSC genome browser views (Fig. 2G, left). Moreover, H3K4me3 levels were low at the ESC-enriched gene, *Pecam1*, in conventional EpiSCs and maEpiSCs (Fig. 2G, top right). Furthermore, maEpiSCs display H3K4me3/H3K27me3 co-occupancy at a custom UCSC browser view of a bivalently marked gene (Fig. 2G, bottom right). Overall, these findings show that maEpiSCs and conventional EpiSCs exhibit similar epigenetic profiles.

### Similar profile of H3K27ac-marked enhancers in maEpiSCs and conventional EpiSCs

**Figure 3:**
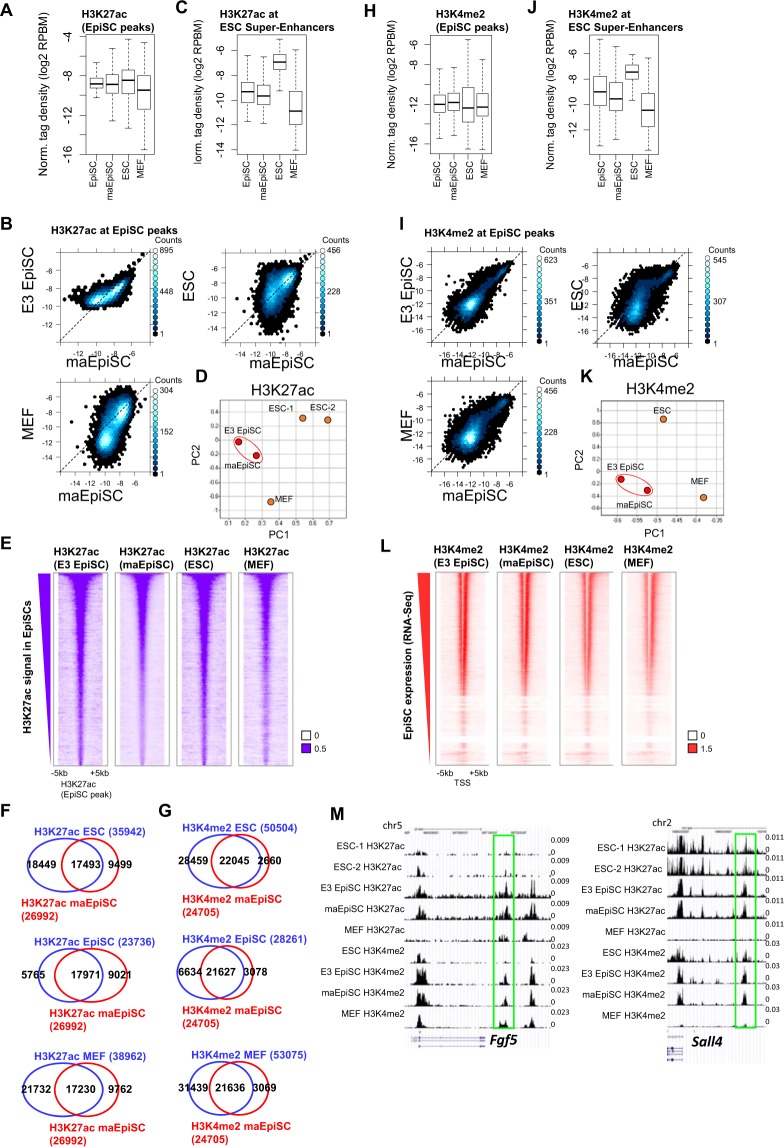
Enhancer profiling of maEpiSCs. (**A**) H3K27ac densities shown using a boxplot and (**B**) scatter plot (normalized tag density; log2 RPBM) at EpiSC-regions in conventional EpiSCs, maEpiSCs, MEFs, and ESCs. (**C**) H3K27ac densities shown using a boxplot at ESC-defined super-enhancers. (**D**) Analysis of H3K27ac levels in maEpiSCs, conventional EpiSCs, ESCs and MEFs using principal component analysis (PCA). (**E**) H3K27ac densities shown in heat maps at EpiSC-defined peaks. (**F**) Overlap between H3K27ac in maEpiSCs and ES cells (top left), maEpiSCs and conventional EpiSCs (middle left), and maEpiSCs and MEFs (bottom left) shown using Venn diagrams. (**G**) Venn diagrams of H3K4me2 levels between maEpiSCs and ES cells (top right), maEpiSCs and conventional EpiSCs (middle right), and maEpiSCs and MEFs (bottom right). (**H**) H3K4me2 densities shown using a boxplot and (**I**) scatter plots at EpiSC-defined regions. (**J**) H3K4me2 density shown using a boxplot at ESC-defined super-enhancers. (**K**) PCA of H3K4me2 levels between maEpiSCs, conventional EpiSCs, ES cells and MEFs. (**L**) Heat maps of H3K4me2 densities at transcriptional start site (TSS) regions. (**M**) Custom UCSC views of H3K4me2 and H3K27ac levels in conventional EpiSCs, maEpiSCs, MEFs, and ESCs.

*Cis-*regulatory elements determine lineage-specific transcriptional responses to external stimuli^18–20^. To evaluate enhancer profiles in maEpiSCs, conventional EpiSCs, ES cells, and MEFs we performed H3K27ac ChIP-Seq, which marks active enhancers^21,22^. We observed similar levels of H3K27ac between conventional EpiSCs and maEpiSCs at EpiSC-peaks (Fig. 3A), while MEFs (GSE29218) displayed lower levels, and ES cells exhibited higher levels, demonstrating that H3K27ac levels at active enhancer regions in maEpiSCs are more similar to conventional EpiSCs relative to ES cells or MEFs. Furthermore, a comparison of maEpiSCs and conventional EpiSCs showed that levels of H3K27ac in intergenic levels are similar (Fig. 3B, left panel). However, a subset of enhancer regions occupied by H3K27ac exhibited elevated levels in conventional EpiSCs, suggesting that a subset of conventional EpiSC-enhancers may have been deactivated in maEpiSCs. In contrast, levels of H3K27ac in intergenic regions were less similar between maEpiSCs and MEFs or ES cells (Fig. 3B, right).

Recent studies have shown that mediator and master transcription factors co-occupy enhancer clusters, also known as ‘super-enhancers’, which have been used to define cellular identity^23,24^. To evaluate the activity of ESC-defined super-enhancers in maEpiSCs, we surveyed H3K27ac levels. These results show that levels of H3K27ac are lower in maEpiSCs relative to ES cells (Fig. 3C), but similar to conventional EpiSCs. These findings likely reflect differential marking of super-enhancers between ES cells and EpiSCs. Also, H3K27ac levels were higher in maEpiSCs relative to MEFs at ESC-defined super-enhancers (Fig. 3C), showing that the activity of ES cell super-enhancers is higher in maEpiSCs relative to MEFs. PCA analysis confirmed that H3K27ac levels at conventional EpiSC peaks were similar between maEpiSCs and conventional EpiSCs relative to ES cells or MEFs (Fig. 3D), where maEpiSCs and conventional EpiSCs clustered closer to one another within the two-dimensional space relative to ES cells or MEFs. In addition, heat maps showed that conventional EpiSCs and maEpiSCs exhibit relatively similar global profiles of H3K27ac (Fig. 3E). Moreover, our results show that 73% of H3K27ac-marked regions in conventional EpiSCs were also marked by H3K27ac in maEpiSCs, whereas 49% of H3K27ac-marked regions in ESCs were occupied by H3K27ac in maEpiSCs (Fig. 3F), and 44% of H3K27ac-occupied regions in MEFs were also occupied by H3K27ac in maEpiSCs (Fig. 3F), suggesting that H3K27ac levels in maEpiSCs are more similar to conventional EpiSCs than MEFs or ESCs.

To further investigate the enhancer landscape of maEpiSC we evaluated H3K4me2 levels, which are also enriched at enhancer regions^25^. Similar to H3K27ac, H3K4me2 levels are correlated with enhancer activity in a cell-type specific manner. Our findings show that 77% of H3K4me2-occupied regions in conventional EpiSCs were also marked by H3K4me2 in maEpiSCs, whereas 44% of H3K4me2-marked regions in ESCs were co-occupied by H3K4me2 in maEpiSCs (Fig. 3G), and 41% of H3K27ac-occupied regions in MEFs were also occupied by H3K27ac in maEpiSCs (Fig. 3G), suggesting that H3K27ac levels in maEpiSCs are more similar to conventional EpiSCs than MEFs or ESCs. In addition, while ChIP-Seq demonstrated that H3K4me2 levels are similar between maEpiSCs and conventional EpiSCs (Fig. 3H), H3K4me2 levels at EpiSC-occupied enhancers were lower in ES cells (GSE53087) and MEFs (GSE36292)^26^, demonstrating that maEpiSCs cells are more similar to conventional EpiSCs than MEFs or ESCs. In addition, levels of H3K4me2 were similar between maEpiSCs and conventional EpiSCs (Fig. 3I, left). However, levels of H3K4me2 were less similar between maEpiSCs and ESCs or MEFs (Fig. 3I, right, bottom), further suggesting that the epigenetic profile of maEpiSCs is more similar to conventional EpiSCs than MEFs or ESCs. Likewise, levels of H3K4me2 at ES cell defined super-enhancers were similar between maEpiSCs and conventional EpiSCs (Fig. 3J). However, H3K4me2 levels were lower in maEpiSCs/conventional EpiSCs relative to ES cells, but higher relative to MEFs at ESC-defined super enhancers (Fig. 3J). PCA analysis confirmed that H3K4me2 levels were similar between maEpiSCs and conventional EpiSCs relative to ES cells or MEFs (Fig. 3K) where maEpiSCs and conventional EpiSCs clustered closer to one another within the two-dimensional space relative to ES cells or MEFs. Heat maps showed that conventional EpiSCs and maEpiSCs exhibit similar global profiles of H3K4me2 relative to ES cells or MEFs (Fig. 3L), where H3K4me2 levels were higher in maEpiSCs and conventional EpiSCs relative to ESCs and MEFs, at genes whose expression is elevated in EpiSCs. Custom genome browser views demonstrate that levels of H3K4me2 and H3K27ac are enriched at pluripotency-associated (*Sall4*) and EpiSC-enriched genes (*Fgf5*) in maEpiSCs (Fig. 3M). Altogether, our results demonstrate that the enhancer landscape of maEpiSCs is similar to conventional EpiSCs.

### maEpiSCs are capable of differentiating

**Figure 4:**
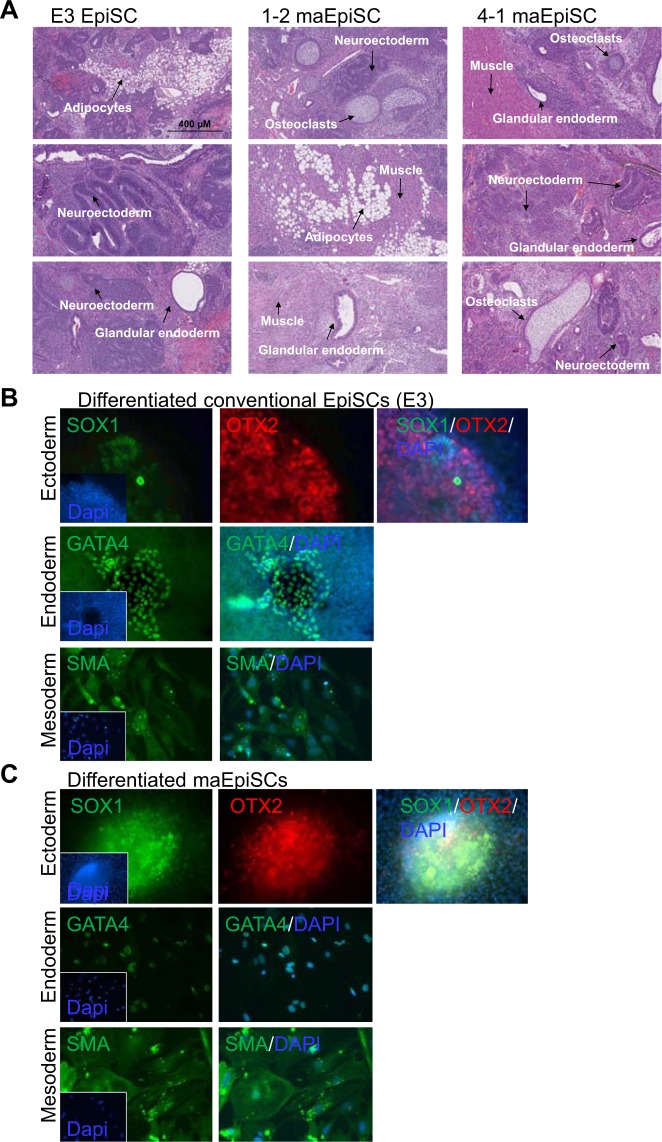
Differentiation of maEpiSCs *in vitro* and *in vivo*. (**A**) Teratomas generated from conventional EpiSCs (left), 1–2 maEpiSCs (middle), and 4-1 maEpiSCs subcutaneously injected into immune compromised mice (SCID-beige). Teratomas were recovered 4–6 weeks following injection and analyzed using standard histological methods (hematoxylin and eosin staining (H&E)). Microscopy (transmitted white-light) of sectioned teratomas. Differentiation of conventional EpiSCs and maEpiSCs into heterogeneous teratomas consisting of endoderm (glandular structures), mesoderm (muscle, adipocytes, and osteoblasts), and ectoderm (neuroectoderm) cells. (**B,C)** *In vitro* differentiation of (**B**) conventional (E3) EpiSCs and (**C**) maEpiSCs in the absence of growth factors into ectoderm (SOX1), endoderm (GATA4), and mesoderm (SMA).

To evaluate the ability of maEpiSCs to differentiate we induced teratoma formation (Fig. 4A), where maEpiSCs or conventional EpiSCs were subcutaneously injected into immune-compromised mice (SCID-beige). maEpiSCs subsequently gave rise to cells found in the three germ layers such as endoderm (glandular endoderm), mesoderm (muscle, fat, osteoclasts, and osteoblasts), and ectoderm (epidermis) (Fig. 4A, middle and right). Likewise, conventional EpiSCs also gave rise to cells found in the three germ layers such as endoderm (glandular endoderm), mesoderm (fat, muscle), and ectoderm (neuroectoderm) (Fig. 4A, left). The ability to generate teratomas demonstrates the functionality of maEpiSCs. Moreover, immunofluorescence analysis showed that *in vitro* differentiated maEpiSCs and conventional EpiSCs express OTX2 and SOX1 (ectoderm), SMA (mesoderm), and GATA4 (endoderm) (Fig. 4B,C). Overall, our findings demonstrate that maEpiSCs are able to differentiate *in vivo* and *in vitro* into cells found in the three germ layers.

## Discussion

Previous studies have demonstrated that ES cells can be derived from haploid embryos^12^. However, EpiSCs have not been directly generated from haploid embryos. Here, we demonstrate that EpiSCs can be generated from haploid embryos. Although we derived maEpiSCs in FGF4-containing media, it is possible that conventional EpiSC media containing bFGF may also support the generation of EpiSCs from haploid embryos. In addition, our results show that established maEpiSCs described in this study, and conventional EpiSCs, contain a diploid genome with a normal number of chromosomes. However, because ploidy analysis was performed after establishment of maEpiSC lines, and not during the initial derivation, it is unclear whether maEpiSCs exhibited a decrease in the percentage of haploid cells during culture, or whether diploidization of cells is required for derivation of maEpiSCs under these conditions. Therefore, results described here do not rule out the possibility that maEpiSCs could be generated and maintained in a haploid state using culture conditions described in this study, or under alternative culture conditions, as it was previously shown that haploid ES cells could be differentiated into haploid EpiSCs^27^. In addition, while we derived maEpiSCs from blastocyst-stage embryos, conventional EpiSCs are derived from E5.5-E6.5 embryos^1,4^. Therefore, it is unclear whether haploid EpiSCs could be derived from E5.5-E6.5 embryos, as previous results demonstrate that haploid ES cells exhibit diploidization upon differentiation^12^.

Moreover, while FGF4 has previously been shown to support the self-renewal of both mouse TS cells and EpiSCs^7,10^, our results demonstrate that the haploid genome of blastocysts favors the generation of EpiSCs over TS cells in FGF4-conditions. Interestingly, while we observed trophoblast giant cells from the outgrowths of haploid blastocysts (Fig. 1D), we did not observe the formation of TS cell colonies, suggesting that formation of TS cells may require a diploid genome.

Our results also show that pluripotent maternal (parthenogenetic/gynogenetic) EpiSCs (maEpiSCs) and conventional EpiSCs exhibit a similarly primed transcriptional profile and epigenetic landscape relative to pluripotent ESCs and differentiated MEFs. We further reveal that the transcriptional profile of maEpiSCs is more similar to conventional EpiSCs than ESCs or differentiated cells such as MEFs or EBs. The transcriptional network intersection between maEpiSCs and conventional EpiSCs may represent a primed-pluripotent gene network. Moreover, the variation in culture conditions between maEpiSCs and conventional EpiSCs represent a resource to identity genes that define the primed-pluripotent network. In this case, alterations in external signals or culture media components, which do not alter self-renewal, may produce transcriptional noise of genes not required for pluripotency. A subset of the stably expressed genes may represent the minimal network of primed-pluripotent EpiSCs.

We also demonstrate that the epigenetic profile of maEpiSCs is more similar to conventional EpiSCs than MEFs or ESCs. These global epigenetic analyses demonstrate similar genome-wide landscapes of active enhancers and histone modifications between maEpiSCs and conventional EpiSCs. In contrast, MEFs and ES cells displayed less similar histone modification profiles. Moreover, enhancer activity, as measured by H3K27ac and H3K4me2 levels, was similar between maEpiSCs and conventional EpiSCs relative to ES cells or MEFs. Our results also demonstrate that super-enhancers are similarly active in maEpiSCs and conventional EpiSCs, and we show that maEpiSCs are fully capable of differentiating *in vitro* and *in vivo*. Altogether, our findings provide a novel method to derive EpiSCs from haploid embryos, and lend epigenetic insight into mechanisms of EpiSC self-renewal.

## Materials and Methods

### Culture of EpiSCs

Conventional EpiSCs (E3) were cultured as previously described^13,28^. Briefly, EpiSCs were cultured on FBS-coated dishes in Knockout DMEM media supplemented with 20% KnockOut serum replacement (SR), penicillin-streptomycin, L-glutamine, and bFGF^13,29^. EpiSCs cells were passaged by washing with Phosphate Buffered Saline (PBS), and dissociating with accutase using serological pipettes (sc-200279; sc-200281). EpiSCs were cultured in the presence of ROCK inhibitor (10 µM, Y27632) for 24 hr following passage.

### Derivation of maEpiSCs

Mature oocytes were isolated from the oviducts of C57Bl/6 J female mice that had been superovulated with 5 IU pregnant mare serum gonadotropin (PMSG) and subsequently 5 IU of human chorionic gonadotropin (hCG) 48 hours later. Cumulus-oocyte complexes (COCs) collected from oviducts were treated with 0.1% hyaluronidase to disperse cumulus cells. Denuded oocytes were activated in M16 media using 5 mM strontium chloride and 2 mM EGTA as previously described^11^, and subsequently cultured to the blastocyst-stage in M16 or KSOM media in microdrops covered by mineral oil. Blastocysts were then transferred to 24-well dishes containing inactivated MEFs and media containing RPMI 1640, 20% FBS, sodium pyruvate, L-glutamine, β-mercaptoethanol (100 µM), pen/strep, FGF4 (25 ng/mL), and heparin (1 µg/mL). maEpiSC colonies were pick and expanded in the same media. Experiments were performed at least three times.

For ChIP-Seq and RNA-Seq experiments, maEpiSCs were transitioned to dishes containing maEpiSC medium, 70% iMEF-conditioned maEpiSC medium, FGF4, and heparin, and were cultured at 37 °C with 5% CO_2_. maEpiSCs were passed twice to remove iMEFs.

### Derivation of TS cells

Trophoblast stem (TS) cells were derived as described previously^7,8,30^. Briefly, male and female C57Bl/6 J mice were mated, and resulting E3.5 blastocysts were flushed in M2 medium. Blastocysts were subsequently transferred to 24-well dishes containing mitotically inactivated MEFs (iMEFs) and media containing RPMI 1640, 20% FBS, 1 mM sodium pyruvate, 100 mM β-mercaptoethanol, 2 mM L-glutamine, recombinant FGF4 (25 ng/mL), and 1 µg/mL heparin.

### Immunofluorescence analysis

Immunofluorescence was performed as previously described with minor modifications^28^. Briefly, to crosslink cells, ESCs were incubated with paraformaldehyde (4%) for 15 min (room temperature), and subsequently washed with 0.1% Triton X-100 three times. Afterwards, cells were blocked for 30 min at room temperature using BSA (1%)/Tween-20 (0.01%) in PBS. Fixed cells were subsequently incubated overnight at 4 °C with a primary antibody (conjugated) in blocking buffer. 12–16 hours later (the next day), cells were washed with blocking buffer (3X) for 15 min.

### Cell cycle profiling

Cell cycle profiling was performed as previously described with minor modifications^28,31^. In brief, cells were harvested by dissociating with 0.25% trypsin, subsequently washed with PBS 1X, and fixed using 70% ethanol. Staining was then performed by treating with a 1 µg/mL DAPI/0.1% Triton X-100 staining solution. The Wayne State University Microscopy, Imaging, and Cytometry core facility assisted with flow cytometry analysis.

### Generation of teratomas

Teratoma formation was performed as previously described with minor modifications^14,28^. Briefly, EpiSCs cells were dissociated into small clusters of cells using Accutase, and one million EpiSCs cells were subcutaneously injected into SCID-beige mice. Mice were subsequently euthanized after three to four weeks. Next, teratomas were washed in PBS (1X) and fixed using formalin (10%) for at least 24 hr. Afterwards, teratomas were embedded in paraffin. Hematoxylin and eosin (H&E) staining was then performed on thinly cut sections using standard techniques. Mice were treated in accordance with the Institutional Animal Care and Use Committee (IACUC) guidelines under approved protocols at Wayne State University.

### ChIP-Seq

ChIP-Seq was performed as previously described^14–17,28,32,33^. Briefly, the H3K4me3 antibody (17–614) was obtained from Millipore, while the H3K27me3 (ab6002), H3K27ac (ab4729), and H3K4me2 (ab32356) antibodies were obtained from Abcam. 15 million mouse ES cells were harvested and fixed with formaldehyde (1%) at 37 °C for 8 min. Sonication was subsequently performed. Cell extracts from 5 million sonicated cells were used for ChIP. Following ChIP, DNA ends were repaired using a End-It DNA End-Repair kit (Epicentre). Next, addition of a single A was administered, and ligation was performed using Illumina-compatible adapters (custom). PCR was then run using a Phusion 2X master mix with high fidelity buffer. Libraries were sequenced on an Illumina HiSeq platform. Bowtie2^34^ was used to map sequenced reads to the mm9 mouse genome using default settings. ChIP-enriched peaks were identified using Spatial clustering for identification of ChIP-enriched regions (SICER)^35^ (SICER settings: 200 bp window size; 400 bp gap setting; 0.001 FDR). The reads per base per million reads (RPBM) measure was used to evaluate ChIP-Seq densities at genomic regions^17,33,36^. R was used to generate boxplots and scatter plots of ChIP-Seq densities.

### RNA-Seq

RNA-Seq transcriptome analysis was performed as described previously^14,15,17,28^. Briefly, mRNA (poly-A) was isolated using a commercial kit (Dynabeads mRNA purification kit). Double-stranded cDNA (dscDNA) was constructed using a commercial kit (Super-Script double-stranded cDNA synthesis kit). Library preparation was performed using fragmented cDNA as described for ChIP-Seq above. Libraries for RNA-Seq were sequenced following the manufacturer’s protocol using an Illumina HiSeq platform.

Reads per kilobase (of exon model) per million reads (RPKM)^37^ was utilized to quantitate gene expression levels of mRNA using RNA-Seq data. edgeR was used to identify differentially expressed genes (fold-change >1.5, FDR <0.001)^38^.
